# Supplementary material for: Modified Natural Dolomite and Its Influence on the Production of Glycerol Carbonate: Effects of Structural and Basicity Properties
Source: Materials (Basel). 2021 May 1;14(9):2358. doi: 10.3390/ma14092358 (PMC8125416; doi:10.3390/ma14092358)
Supplement: Supplementary file 1 [file materials-14-02358-s001.zip › materials-1175051-supplementary.pdf]

materials

Supplementary material

Modified Natural Dolomite for the Catalytic Production of Glycerol Carbonate: Effects of Structural and Textural Properties

Julio González-García [1,2,*], Lifang Chen [3,*], Omar Campuzano-Calderon [1], Sara Núñez-Correa [1,4], Enrique A. López-Guajardo [1], Jin An Wang [3] and Alejandro Montesinos-Castellanos [1,*]

1 Tecnologico de Monterrey, Escuela de Ingeniería y Ciencias, Ave. Eugenio Garza Sada 2501, Monterrey, 64849 Nuevo León, Mexico; a00824173@itesm.mx (O.C.-C.); sarnunez@uv.mx (S.N.-C); enrique.alopezg@tec.mx (E.A.L.-G.)
2 Tecnologico de Estudios Superiores de Coacalco. 16 de septiembre 54, Col. Cabecera municipal, Coacalco de Berriozabal, 55700 Estado de México, Mexico.
3 ESIQIE, Instituto Politécnico Nacional, Av. Instituto Politécnico Nacional s/n, Col. Zacatenco, 07738 Ciudad de México, Mexico; jwang@ipn.mx
4 Facultad de Ciencias Químicas, Universidad Veracruzana, Campus Coatzacoalcos, 96538 Veracruz, Mexico.
* Correspondence: jgon88@hotmail.com (J.G.-G.); lchen@ipn.mx (L.C.); alejandro_montesinos@tec.mx (A.M.-C.)

Table S1. Atomic Fractional Coordinates of the different Crystalline Structures.

Compound	Atom	Site	x	y	z
CaO	Ca	4a	0.0000	0.0000	0.0000
	O	4b	0.5000	0.5000	0.5000
$Ca(OH)_2$	Ca	1a	0.0000	0.0000	0.0000
	O	2d	0.3333	0.6667	0.2346
	H	2d	0.3333	0.6667	0.4280
MgO	Mg	4a	0.0000	0.0000	0.0000
	O	4b	0.5000	0.5000	0.5000
$CaCO_3$ (Trigonal R-3c:H)	Ca	6b	0.0000	0.0000	0.0000
	C	6a	0.0000	0.0000	0.2500
	O	18e	0.2500	0.0000	0.2500
$CaCO_3$ (Orthorhombic Pbnm)	Ca	4a	0.0000	0.0000	0.0000
	C	4c	0.0794	0.6460	0.2500
	O1	4c	0.3722	0.5890	0.2500
	O2	8d	-0.0552	0.6678	0.1219
$CaCO_3$ (Orthorhombic Pmcn)	Ca	4c	0.2500	0.4141	0.7538
	C	4c	0.2500	0.7662	0.0000
	O1	4c	0.2500	0.9187	0.0000
	O2	8d	0.4733	0.6837	0.0000
$CaSO_4$	Ca1	4c	0.7500	0.0000	0.3460
	S1	4c	0.2500	0.0000	0.1550
	O1	8g	0.2500	0.1710	0.0150
	O2	8g	0.0800	0.0000	0.2980
$Ca_2P_2O_7$	Ca1	4a	0.1374	0.2313	0.0000
	Ca2	4a	0.1591	0.5447	0.2426
	Ca3	4a	0.7895	0.7337	0.1380
	Ca4	4a	0.3627	0.1054	0.1355
	P1	4a	0.6213	0.3078	0.0235
	P2	4a	0.8673	0.2534	0.1243
	P3	4a	0.0463	0.7148	0.0186
	P4	4a	0.2873	0.6462	0.1207
	O1	4a	0.4607	0.1584	0.0426
	O2	4a	0.7727	0.1984	-0.0133
	O3	4a	0.5303	0.4921	-0.0001
	O4	4a	0.7395	0.3776	0.0789
	O5	4a	0.7364	0.0873	0.1469
	O6	4a	0.0519	0.1769	0.0953
	O7	4a	0.8994	0.4088	0.1695

	O8	4a	0.8537	0.7907	0.0433
	O9	4a	0.1855	0.8795	-0.0011
	O10	4a	0.0229	0.5542	-0.0249
	O11	4a	0.1582	0.5873	0.0666
	O12	4a	0.3450	0.4526	0.1474
	O13	4a	0.4622	0.7756	0.1015
	O14	4a	0.1474	0.7722	0.1572
$Mg_2P_2O_7$	Mg1	8j	0.2496	0.925	0.1212
	Mg2	8j	0.6986	0.433	0.8286
	P1	8j	0.9479	0.764	0.7649
	P2	8j	0.5245	0.773	0.4683
	O1	8j	0.729	0.833	0.5901
	O2	8j	0.3762	0.761	0.5513
	O3	8j	1.1276	0.769	0.6999
	O4	8j	1.0113	0.907	0.8953
	O5	8j	0.9236	0.596	0.8287
	O6	8j	0.471	0.897	0.3203
	O7	8j	0.5998	0.606	0.4192

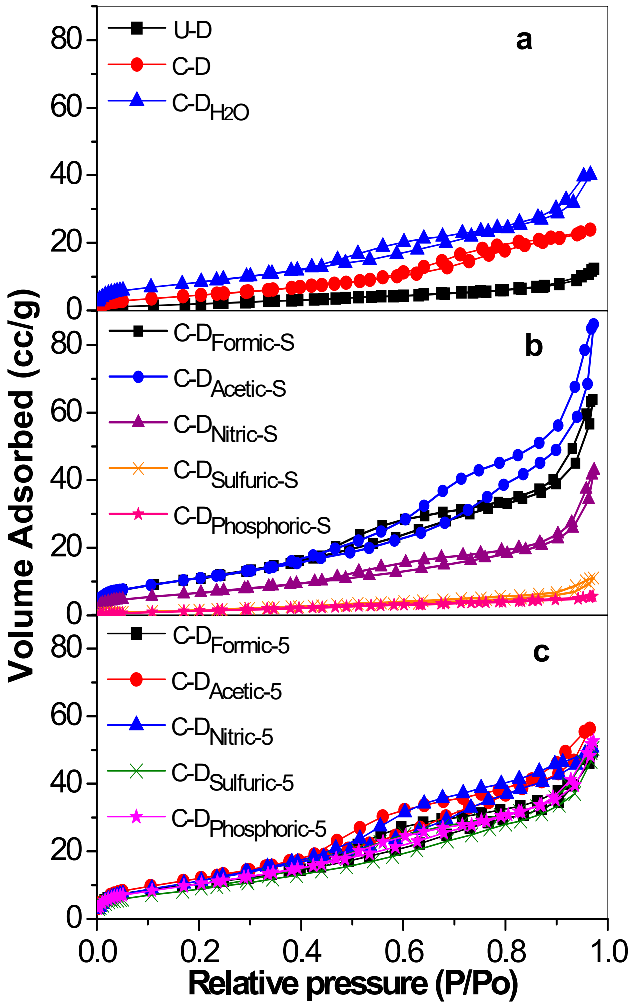

Figure S1. N_2 adsorption-desorption isotherms of (**a**) samples U-D, C-D, and C-D$_{H_2O}$, (**b**) calcined samples treated by stoichiometric ratios of different acids, and (**c**) samples treated at pH =5.
